# Screening of Group B *Streptococcus* in pregnancy: A systematic review for the laboratory detection

**DOI:** 10.1515/med-2025-1197

**Published:** 2025-08-11

**Authors:** Valentina Arsić Arsenijević, Vladimir Gerginić, Biljana Miličić, Aleksandar Jurišić, Ljubomir Petričević

**Affiliations:** Institute of Microbiology and Immunology, Medical Mycology Reference Laboratory (MMRL), University of Belgrade Faculty of Medicine, Dr Subotića 1, Belgrade, 11000, Serbia; Medical School, Narodni Front University Hospital, University of Belgrade, Belgrade, Serbia; Department of Medical Statistics and Informatics, School of Dental Medicine, University of Belgrade, Belgrade, 11000, Serbia; Department of Obstetrics and Gynecology, Medical University of Vienna, Vienna, Austria

**Keywords:** *Streptococcus* group B, pregnancy, systematic review, laboratory tests

## Abstract

**Background:**

Group B *Streptococcus* (GBS) is important since almost 1/3 of pregnant women are colonized with GBS, and as much as 50% passes to the newborns, sometimes resulting in severe neonatal infections; that is why there are mandatory guidelines for antepartum screening for GBS vaginal/rectal colonization. Also, bacteria other than GBS and yeasts may affect newborns; therefore, an increase in the current knowledge and improving the guidelines related to the prediction and prevention of neonatal early-onset infections are needed.

**Methods:**

A systematic review was performed to investigate risks, types of specimens, sampling methods, media for GBS recovery, identification tests, gestation week for testing, GBS prevalence, sensitivity, specificity, turnover time for cultures, antigen, and molecular-based tests. A literature search was conducted through the Web of Science, Scopus, and PubMed.

**Results:**

A total of 20 studies were identified with 10,288 patients and 1,334 GBS positive (13%). Eight studies were performed in adequate gestation week and revealed prevalence from 0.2 to 20.8% (conventional tests) and 37 to 45% (molecular tests). In only three studies, vaginal/rectal swab recommended by guidelines was applied.

**Conclusions:**

The heterogeneity of the detection and identification of GBS reduces the scientific and clinical utility of laboratory-based data, and universal antepartum screening with affordable, high-sensitivity traditional tests is needed.

## 1 Introduction

The vaginal microbiome consists of an ecological community of microorganisms that are important for both maternal and neonatal health. During pregnancy, the vaginal microbiome composition changes, which has a role in ascending infections. For neonates, exposure to the vaginal microbiome during birth or through premature rupture of membranes is an important route for early-onset neonatal infections (EONI). *Streptococcus agalactiae*, commonly known as Group B *Streptococcus* (GBS), is a leading cause of EONI. Therefore, carriage investigation among pregnant women by using a screening-based strategy provides the most definitive overarching evidence for clinicians and healthcare staff to prevent the potential harms of GBS infection in newborns timely. While often residing asymptomatically in healthy individuals and can colonize the gastrointestinal and genitourinary tract, in some conditions, GBS might cause urinary tract or skin and soft tissue infections in adults. Up to 1/3 of pregnant women is colonized with GBS (10–40%), and 50% of them may transmit it to the newborn, so adhering to GBS screening guidelines to prevent neonatal infection is important but overall findings demonstrate an averaging low rate of compliance [1]. Severe and life-threatening complications, such as pneumonia, sepsis, or meningitis, are more common in neonates in undeveloped countries, and several guidelines were created with the goal of preventing GBS-related diseases timely [2].

Substantial progress of perinatal GBS screening is done since the first guideline published in 1996, and revised by CDC in 2010 [3]. The stewardship of the guideline was transferred to professional organizations, so The American College of Obstetricians and Gynecology created a recommendations for prophylaxis and treatment of GBS [4], and the American Society for Microbiology created a recommendations for standard laboratory practices related to GBS detection and identification [5]. This universal antepartum GBS screening at 35–37 weeks of gestation, chemoprophylaxis during childbirth, and management of newborns aim to achieve the best neonatal outcomes.

Although much progress has been made, important challenges from a laboratory perspective remain, mainly focusing on proper types of specimens, collection methods, incubation, quantification and identification, and application of molecular, non-culture-based tests for GBS recovery. Deeper insight into molecular tests, their sensitivity, and specificity in routine screening seems important to improve the practice and reduce the rate of GBS neonatal infection, but also to extend it to a symptom-driven approach that groups probable pathogens into one cost-effective and accurate tests in a clinically relevant timeframe.

Although universal screening has been important for the reduction of EONI, some data present that these recommendations are not equally adopted worldwide, and this systematic review aimed to perform the analysis of the current application of the existing guidelines for GBS detection and identification by gathering laboratory data regarding types of specimens, sampling methods, media for GBS recovery, identification tests, gestation week for testing, and GBS prevalence. In addition, sensitivity, specificity and turnover time for culture tests, antigen (Ag) tests, and molecular-nucleic acid amplification tests (NAATs) review, including differential media for GBS recovery by culture, polymerase chain reaction (PCR) for DNA detection, and matrix-assisted laser desorption/ionization time-of-flight mass spectrometry (MALDI-TOF MS) for accurate GBS identification.

## 2 Materials and methods

This systematic review of literature was conducted according to the guidelines of the Preferred Reporting Items for Systematic Reviews and Meta-Analyses (PRISMA) [6].

### 2.1 Search strategy

We aim to identify all types of studies that examined an association between GBS infections in pregnancy, laboratory-based variables, and risk factors for infection and outcomes of pregnancy. Several international and regional databases were searched systematically.

**Figure 1:**
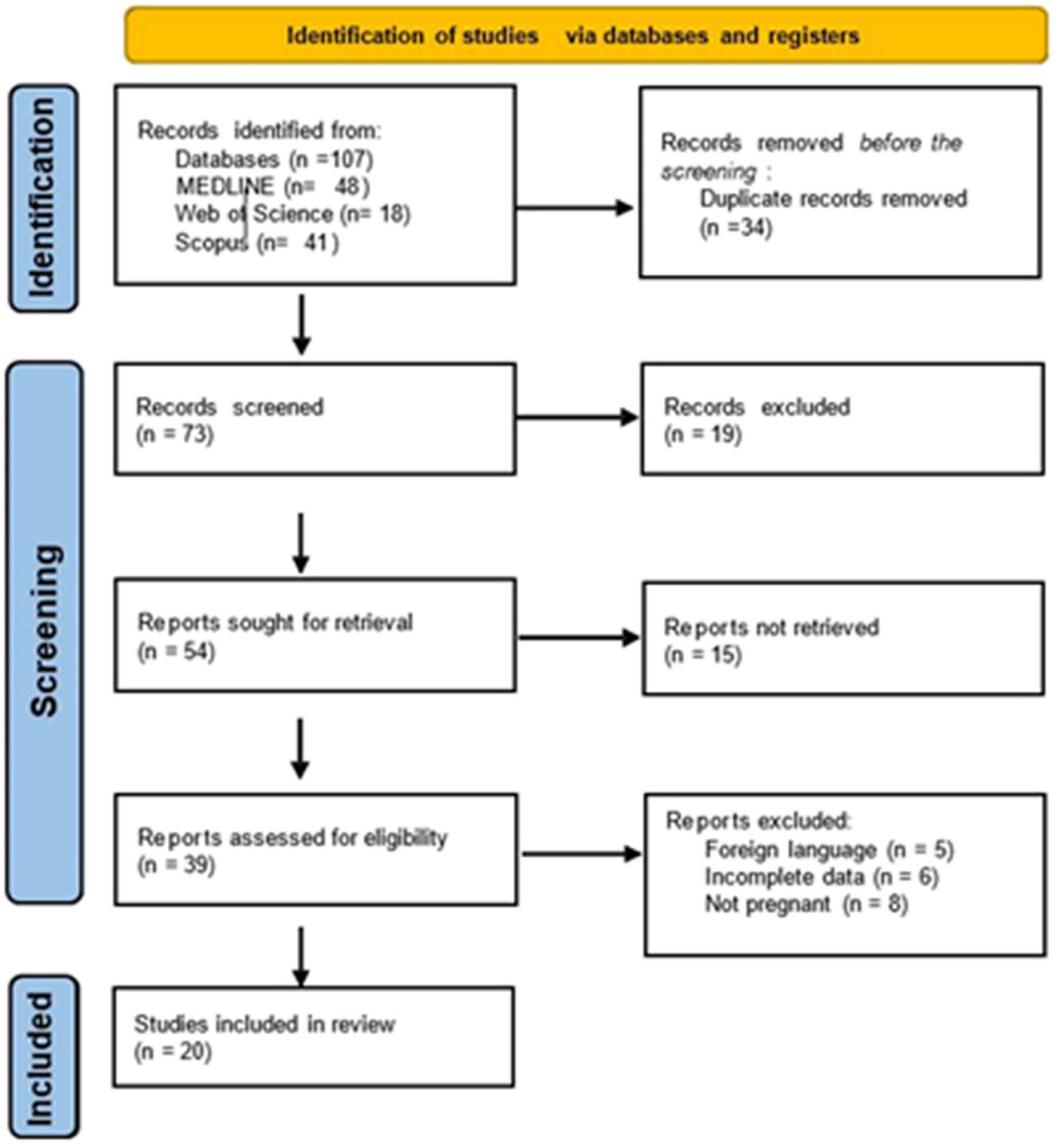
GBS and pregnancy – PRISMA flow chart of publication selection for systemic literature review.

The electronic search was performed in July 2023 in databases: MEDLINE (from 2003 to the present), Web of Science (from 2003 to the present), and Scopus, thus, making a 20-year survey on the published papers. We used the search strategy as a combination of keywords such as controlled vocabulary (MESH – in upper case) and free text terms (in lower case): ((VULVOVAGINITIS) OR (VAGINITIS)) OR (vaginal)) OR (vulvovaginal)) AND ((STREPTOCOCCUS GROUP B) OR (STREPTOCOCCUS AGALACTIAE))) AND ((PREGNANCY) OR (PREGNANT WOMEN))) AND (species)) AND (((prevalence) OR (epidemiology)) OR (rate) OR (guideline)). The PRISMA flowchart synthesizes the screening and selection processes (Figure 1).

PICO (P – patient or population; I – intervention or indicator; C – comparison or control; O – outcome) process was used in evidence-based practice to frame and answer a clinical question in terms of the specific patient’s problem. The conducted systematic review of studies assessed the prevalence of maternal GBS colonization and infections in pregnant women at different gestation weeks (P), with the evaluation of different laboratory tests for GBS detection and determination (I). Screening and treatment for GBS were analyzed in relation to identifying risk factors that may influence the prevalence of GBS, like ethnicity, smoking, and maternal age (C). Clinical outcomes (O) were GBS positivity, analyzed in relation to sampling procedure, identification method, and potential etiologies associated with GBS infection/colonization.

To assure the reliability of the data collected, the electronic search was further supplemented with additional citation searching through the reference lists of identified studies and relevant reviews.

### 2.2 Eligibility criteria

Original articles that were included in this review were prospective, cross-sectional, or retrospective studies by study design. Studies on animals, *in vitro* cultures, abstracts, papers in non-English language and articles with inadequate sample or incomplete data were excluded.

#### 2.2.1 Population

Pregnant women in different gestation weeks screened for GBS were included in this study. Only those after the 35th week were discussed.

#### 2.2.2 Types of outcome

The outcome variables evaluated in the included studies were patients’ samples, tests for GBS detection and identification, the gestation week for screening GBS prevalence in pregnancy, predisposing factors, and GBS prevalence.

### 2.3 Study selection and data extraction

The selection of studies was performed by two authors (V.G. and A.J.) who initially read the titles and abstracts, and then the studies that met the inclusion criteria were considered in full text. Extracted data were collected by two authors (Lj.P. and B.M.) and supervised by the third author (V.A.A.), and included: author, year of publication, country, sample, GBS positivity, tests for GBS detection and identification, type of study, clinical setting, patients’ characteristics and gestation week, as well as predisposing factors.

Based on the PICO question the inclusion criteria can be summarized as (1) Participants/population: pregnant women with informed consent, aged 18 years or older, in all three trimester, and absence of serious organic or systemic diseases (such as coronary heart disease, stroke, and leukemia). (2) Intervention(s): different laboratory tests for GBS detection and determination. (3) Comparator(s)/control: pregnant women without clinical signs and symptoms of GBS infection and without observed risk factors. (4) Study design: human cross-section studies written in English. To ensure that all relevant clinical information, often not tested in experimental studies, was captured, longitudinal observational studies (retrospective and prospective comparative cohort and case–control studies) were also included. (5) Main outcome(s): the primary outcomes of concern were GBS detection.

Based on the PICO question the exclusion criteria can be summarized as (1) papers presenting repeated results or were retracted, reviews, meta-analyses, meeting abstracts, case reports, laboratory or animal studies, editorials, or letters; (2) studies without a direct comparison between groups; and (3) studies published in languages other than English.

### 2.4 Methodological quality assessment criteria for the evaluation of eligible studies

The Joanna Briggs Institute (JBI) critical appraisal tools (checklist for analytical cross-sectional studies) were used to assess the quality of the included studies and possible risk of bias [7]. This tool consists of eight domains related to clear inclusion criteria, detailed setting description, valid/reliable exposure, objective/standard measurement criteria, confounding factor identification, dealing strategies for confounding factors, valid, reliable outcome measurement, and appropriate statistical analysis. The possible answers for the evaluation were *Yes*, *No*, *Unclear*, or *Not/Applicable*. Two reviewers (B.M. and V.G.) independently assessed titles and/or abstracts of citations identified against the eligibility criteria and the quality of studies included. In case of disagreement, a third opinion (V.A.A.) was sought.

## 3 Results

### 3.1 Study characteristics

The number of studies identified through the selection process was 20 (Figure 1), which examined the occurrence of GBS in pregnant women detected during different procedures or protocols. In geographical terms, data were collected from Europe [8–11], Asia [12–17], North America [18,19], and Africa [20–27] (Table 1). The greatest number of patient samples were collected in Japan (*n* = 1.226) [15] and South Africa (*n* = 1.404) [23], while the lowest was in Sri Lanka (*n* = 100) [13] and Tanzania (*n* = 90) [26]. Study design was not explicitly declared in ten cases (50%). In five cases, the study was prospective, in four cross-sectional and two retrospective (Table 1).

**Table 1:** Characteristics of included studies by country: predisposing factors, positive GBS findings, sampling type, detection, and identification methods

Country	Positive GBS (%)	Sampling type	Test for detection	Type of study	Settings	Predisposing factors/risks
Year	All infections		Test for identification		Patients	
[Ref.]			AST		Gestation week	
**Europe**						
Italy	48/245 (19%)^#^	Vaginal swab	Culture BA	Retrospective	Hospital	NA
2021	245/245		AST		Premature delivery	
[8]					24–36	
Italy	265/388 (NA)	Vaginal swab	Culture	Prospective	Hospital	NA
2012	388		ChromID Strepto B agar		All pregnant patients	
[9]			Surface molecules PCR		NA	
			Multilocus sequence typing (serotype V)			
Denmark	117/668 (18%)	Vaginal swab	Culture BA	NA	Clinical research unit	NA
2014	644/668	Fornix posterior – thigh			Healthy pregnant	
[10]*					36	
France	32/190 (16.8%)	Vaginal swab	Culture, Columbia BA, Chromogenic StrepBt (BioRad), Medium Granada (bioMérieux),	NA	NA	NA
2009	NA				Pregnant women	
[11]					NA	
**Asia**						
India	25/524 (4.8%)^#^	Vaginal swab	Culture – aerobic	Cross-sectional	Hospital	Ethnicity previous labors smokers
2010	NA	fornix posterior – thigh			Pregnant women admitted at term and in preterm labor	Gestational age preterm labor
[12]					NA	
Sri Lanka	18/100 (18%)	Vaginal swab introitus vagine (low)	Culture	Cross-sectional	Teaching hospital	NA
	Conventional					
2021	45/100 (45%)	Rectal swab	Real-time PCR		Pregnant women	
	PCR					
[13]*	NA				>35	
China	NA	Vaginal swab	PCR	NA	Municipal hospital	Gestational diabetes mellitus
2022	NA				Spontaneous preterm birth and control	
[14]					First or early second trimester	
Japan	154/1226 (12.6%) BA	Vaginal swab	Culture (Todd-Hewitt broth, BA)	NA	University hospital pregnant women	NA
2015	192/1226 (15.7%) PCR		PCR (dltS and cps genes)		36–39	
[15]*	NA		Types Ia, Ib, III			
Japan	48/583 (8.2%)	Vagina swab	Culture (Todd-Hewitt broth, BA)	NA	Medical Center	NA
2003	NA	fornix posterior (high)	CPS antigens		Pregnant women	
[16]			Types VIII, VI, Ib, III		28	
China	190/1391 (13.7%)	Vaginal rectal swab	Culture (enriched broth media)	Prospective	General Hospital	NA
2023	15/190^$^ (7.9%)		BA, Chrom agar		Pregnant women	
[17]*	NA		CAMP test		35–37	
			VITEK-2, MALDI-TOF MS			
**North America**						
Canada	102	Vaginal rectal swab	Culture (BA)	Prospective	Hospital	NA
2017			Multilocus sequence typing (serotype III, Ia, V)		Healthy pregnant women	
[18]			PCR			
USA	150^#^	Vaginal swab	Histone deacetylase	NA	Hospital	NA
2019			Sequences amplified from the V1–V3 region of bacterial ribosomal 16S rRNA genes – PCR		First trimester	
[19]					NA	
**Africa**						
Sudan	16/200 (8%)	Vaginal swab Cervical swab	Microscopy	Cross-sectional	Hospital	NA
2014	176/200 (88%)		Culture (BA)		Pregnant women	
[20]			Identification: conventional and biochemical		Second and third trimester	
Uganda	3/1472 (0.2%)^#^	Vaginal swab	Culture: selective media	Cross-sectional	Health Centre	NA
2020	955/1472		Identification: biochemical, VITEK-2		HIV-1 negative pregnant women	
[21]*					During labor	
Egypt	17/310 (5.5%)^#^	Vaginal swab	Microscopy	NA	University Hospital Pregnant with vaginitis	NA
2022	211/310		Culture		NA	
[22]			PCR			
South Africa	20/1404 (2.8%)	Vaginal swab	Microscopy	Prospective	NA	HIV does not have an impact on GBS colonization
2011	339/1404		Culture		HIV-positive and negative pregnant women	
[23]*	HIV+ 10/716				During early labor	
	HIV− 10/688				≥36	
	251/716					
	188/688					
Togo	0/302 (0%)^#^	Vaginal swab	Microscopy	Prospective	Hospital	NA
2013	221/302		Culture		Pregnant women	
[24]					NA	
Lebanon	46/221 (20.8%)^#^	Vaginal swab	Culture (BA)	NA	University Hospital Pregnant women	NA
2019	83/221				35–37	
[25]*						
Tanzania	22/90 (24.4%)	Vaginal swab	PCR	NA	NA	NA
2022	NA				During pregnancy	
[26]					two times <20 and ≥20	
Namibia^1^ and South Africa^2^	72/530^1^ (13.6%^1^)	Vaginal/rectal swab	Culture (Todd Hewitt broth, BA)	NA	Hospital	NA
2019	NA		scpB gene for capusle types (II, III, V, Ia, IV)		Pregnant women	
[27]*	37/100^2^ (37.0%)				35–37	
	NA		PCR			

GBS – group B *Streptococcus*; BA – blood agar; MALDI-TOF MS – matrix-assisted laser desorption ionization-time of flight mass spectrometry; NA – not applicable; *publications corresponding to the adequate gestation week for screening; AST – antibacterial susceptibility testing; ^$^CAMP negative GBS; ^#^in combination with species, e.g., *Candida*, *Lactobacillus, S. aureus*, *Enterococcus*, *E. coli*, *Mycoplasma, Ureaplasma*, *Chlamydia*, *Neisseria*, *T. vaginalis, K. pneumonia*.

### 3.2 GBS prevalence and applied tests

Out of a total of 10,288 examined patients, 1,334 were found to be positive for GBS, which is around 13% (Table 1). The tests used for GBS detection were mainly conventional (microscopy and culturing) and were done by swabbing the vaginal mucosa. The sample culturing was dominant (17 publications, 85%), but the exact type of culturing media/agar was reported only in eight (40%) publications (Table 1). In certain studies (*n* = 2), microscopy was paired with cultivation, while in a single publication [22], microscopy was used for GBS detection. In only three studies, PCR was applied [14,19,26], and in five studies, PCR was compared with conventional tests [9,13,15,18,22,27]. Mainly, the GBS identification was done conventionally and in a single study by MALDI-TOF MS [17]. Only eight studies corresponded with the adequate gestation week for screening, showing prevalence from 0.2 to 20.8% (conventional tests) and 37 to 45% (molecular tests).

GBS serotypes were determined in five studies [9,15,16,18,27], and data showed domination of type III [15,16,18,27], followed by type V [9,18,27]. The vaginal microbiota and other infective agents were examined in five studies and revealed different bacteria, mainly *Lactobacillus, Staphylococcus aureus*, *Enterococcus*, *Escherichia coli*, *Mycoplasma, Ureaplasma*, *Chlamydia*, *Neisseria*, *Klepsiella pneumoniae,* as well as yeast *Candida* and protozoa *Trichomonas vaginalis* [8,12,22,24,25].

### 3.3 Patient samples, risks, and predisposing factors

Different sampling methods were used, such as swabs from vaginal/rectal mucosa, with swabbing being a dominant one. The sample was usually taken from the vaginal mucosa during the control visits to the gynecologist, while rarely included sampling during early labor [23]. Sample collection from pregnant women was done after 35 weeks of pregnancy in seven (33.3%) studies, while in others, either this data was not reported or was done in other periods of pregnancy (Table 1). Only in two studies [8,12] samples were taken from patients undergoing premature labor. Two studies confirmed that GBS colonization is associated with the development of postpartum infections [23] and adverse pregnancy outcomes [27]. In only four studies, vaginal/rectal swabs, recommended by guidelines, were applied [13,17,18,27].

Data regarding risk factors were not shown in 18 studies. Only the study from India [12] revealed predisposing factors such as smoking (4.7%), previous childbirths, gestational age (4.7%), and preterm labor (4.7%). HIV positivity does not have an impact on GBS findings [23].

### 3.4 Guidelines recommendation and selected studies

A small number of studies [10,13,15,17,23,25,27] could be used for adequate systematic review since only in those studies did the selection criteria follow the guidelines for GBS screening in pregnancy [3–5]. The selected publications encompassed 7,012 subjects, out of which 604 were GBS positive, representing around 75% of the reported total sample and 50% of all positive GBS (Table 1). These data give the prevalence of GBS around 8.6%. Furthermore, GBS screening method in the selected studies did not give more precise methods than those given for the entire study (previously described).

### 3.5 Quality assessment of included studies

The quality assessment of included studies is presented in Table 2. Two reviewers independently assessed titles and/or abstracts of citations identified by the eligibility criteria and the quality of studies included. In case of disagreement, a third opinion was sought. Out of 20 included studies, six studies had the maximum number of positive responses according to the score used to assess the quality of studies (Table 2) [10,13,21–23,26], two studies had 7 out of 8 positive criteria [12,20], and the remaining 12 studies had 6 out of 8 positive criteria from the quality assessment [8,9,11,14,16–18,24–27]. The common lack of studies that did not have the maximum number of points refers to the lack of analysis of confounding factors, i.e., lack of analysis of risk factors and determination of risk groups for the existence of GBS infection in pregnant women. Also, the authors excluded potentially confounding groups and performed adequate statistical analysis. Therefore, the overall quality of the evidence for this study was considered “Good.” The absence of this type of analysis is also due to the weakness in the quality of the studies, even in studies with a large number of positive evaluations, since this leads to a lack and weakness of monitored outcomes.

**Table 2:** Quality assessment of included studies (JBI cross-sectional studies checklist)

Studies	Clear inclusion criteria	Detailed setting description	Valid/reliable exposure	Objective/standard measurement criteria	Confounding factor identification	Dealing strategies for confounding factors	Valid reliable outcome measurement	Appropriate statistical analysis	Quality score
8	Yes	Yes	Yes	Yes	No	No	Yes	Yes	6/8
9	Yes	Yes	Yes	Yes	No	No	Yes	Yes	6/8
10	Yes	Yes	Yes	Yes	Yes	Yes	Yes	Yes	8/8
11	Yes	Yes	Yes	Yes	Yes	No	Yes	Yes	6/8
12	Yes	Yes	Yes	Yes	Yes	No	Yes	Yes	7/8
13	Yes	Yes	Yes	Yes	Yes	Yes	Yes	Yes	8/8
14	Yes	Yes	Yes	Yes	Unclear	Unclear	Yes	Yes	6/8
15	Yes	Yes	Yes	Yes	No	No	Yes	Yes	6/8
16	Yes	Yes	Yes	Yes	No	No	Yes	Yes	6/8
17	Yes	Yes	Yes	Yes	No	No	Yes	Yes	6/8
18	Yes	Yes	Yes	Yes	No	No	Yes	Yes	6/8
19	Yes	Yes	Yes	Yes	No	No	Yes	Yes	6/8
20	Yes	Yes	Yes	Yes	Yes	No	Yes	Yes	7/8
21	Yes	Yes	Yes	Yes	Yes	Yes	Yes	Yes	8/8
22	Yes	Yes	Yes	Yes	Yes	Yes	Yes	Yes	8/8
23	Yes	Yes	Yes	Yes	Yes	Yes	Yes	Yes	8/8
24	Yes	Yes	Yes	Yes	No	No	Yes	Yes	6/8
25	Yes	Yes	Yes	Yes	No	No	Yes	Yes	6/8
26	Yes	Yes	Yes	Yes	Yes	Yes	Yes	Yes	8/8
27	Yes	Yes	Yes	Yes	No	No	Yes	Yes	6/8

### 3.6 Sensitivity, specificity, and turnover time for cultures, Ag and molecular (NAATs) based tests used for GBS screening

Non-culture-based tests for GBS detection using Ag detection and molecular-based tests, especially NAATs, have increased in recent years [28–34]. Table 3 contains the performance methods, i.e., culture, Ag, and molecular base tests, their sensitivity, specificity, turnaround time, and roughly estimated cost [28–34]. Data presented were compared to the culture technique [28,29,31] taken as a golden standard for GBS detection. Ag-based tests were found to be very specific and highly sensitive at a low cost [28,29]; however, in a large number of PRISMA-selected studies (Figure 1) no Ag tests for GBS were found [8–27]. Molecular-based tests for GBS screening [28–34] proved high sensitivity, the possibility to detect small quantities of bacterial DNA or RNA, the short turnover time within hours, and potential to detect polymicrobial pathogens or pathogens that are not easy to recover by culture. However, there are also some disadvantages to these methods: false-positive results, absence of antibiotic susceptibility test results, high cost, and necessity of expensive equipment.

**Table 3:** GBS detection and the antepartum screening for vaginal–rectal colonization: sensitivity, specificity, and turnover time for cultures, antigens (Ag), and molecular based – NAATs (PCR). Presented data for sensitivity are calculated with culturing taken as the golden standard test

Type of media	Sensitivity (%)	Specificity (%)	Turnaround time (h)	Estimated costs	Ref.
Culture-based					
Culture BA	42.3	100	48–72	Low	[28]
Culture BA	81.5	100	24–48	Low	[29]
Liquid chromogenic medium	71.1	98.1	24–48	Low	[29]
Chromogenic agar plate with pre-enrichment	70.6	91.5	48–72	Low	[29]
Culture*	97.7	100	48	Low	[30]

Type of test	Sensitivity	Specificity	Turnaround time	Cost	Ref.
Antigen based					
GBS Ag PathoDx	57.3	99.5		Low	[28]
GBS Ag (W)	18.5	89.4	0.5–1	Low	[29]
GBS Ag (H)	21.7	74.0	0.5–1	Low	[29]
GBS Ag (W) with pre-enrichment	76.5	96.6	24–48	Low	[29]

Type of test	Sensitivity	Specificity	Turnaround time	Cost	Ref.
Molecular based – NAATs					
PCR scpB	99.6	100	1–2	High	[28]
PCR cfb	75.3	100	1–2	High	[28]
LAMP	100	94.0	0.5–1	High	[29]
LAMP PlusLife^®^ GBS	98.7	92.9	1–2	High	[30]
LAMP Ampliflash^®^ GBS	87.4	100.0	1–2	High	[30]
qPCR Xpert GBS/CE	62	76	1–2	High	[31]
LAMP Ampliflash^®^ GBS	98.1	100	1–2	High	[32]
Ct 33 or 40qPCR					
qPCR Xpert GBS/CE	95.8	64.5	1–2	High	[33]
qPCR Xpert GBS/CE	85.7	95.6	1–2	High	[34]

CE – combined enrichment, ^#^LIM broth is a selective medium for the enrichment of GBS from vaginal and rectal samples (Todd-Hewitt broth); BA – blood agar; LAMP – loop-mediated isothermal amplification; *Columbia blood agar plates 5% horse blood (5% CO_2_) and Granada plates (anaerobic), NAATs – nucleic acid amplification tests.

## 4 Discussion

From the GBS screening-based perspective, for accurate results, properly addressing gestation week, sample type, and laboratory tests, which have high sensitivity and specificity, are very important, and the collected data in the present study for 17 countries contribute to this pool of knowledge (Table 1). The overall findings demonstrate an averaging low rate of compliance with screening protocol, especially for the type of specimens and gestation age. Despite progress in recommendations for universal GBS screening in pregnancy in the present survey, a low number of publications dealing with this topic in the last 20-year period were detected. A total of 20 studies identified 10,288 examined patients and 1,334 positive for GBS (13%), but only in seven studies did testing correspond with the adequate gestation week for screening [10,13,15,17,23,25,27] (Table 1). Pure adherence to universal screening of pregnant women with vaginal–rectal cultures was described in several publications, and these findings led to recognizing the need to develop improved strategies for optimizing antenatal GBS screening adherence [1]. Data from Greece publish the overall maternal colonization rate of 9.6% and discomfort associated with rectal swabbing [33]. The exact reason for low adherence, discomfort associated with vaginal–rectal sampling, is stressed out in other studies. These data showed that apart from less discomfort, the use of vaginal–perineal samples for assessment of maternal GBS colonization is comparable to the recommended vaginal–rectal swab [34], but the broader application of this modification is not straightforward to know.

The reviewed studies provide evidence that the type of sample is important for the success of GBS screening and accurate prevalence rate. Different samples (e.g., vaginal/rectal, vaginal/perianal, only vaginal, and vaginal/cervical) were used for GBS detection in analyzed studies despite the strict recommendation [5]. In only four studies recommended type of sample (vaginal/rectal) was applied [13,17,18,27] and done correctly starting from the vagina, by entering 2 cm above the introitus, then over the perineum region to the rectum and up to 1 cm into the rectum (Table 1). In this survey, the importance of dual sites testing (vaginal/rectal) was clearly demonstrated only by Mukesi [27], and data reported from Namibia demonstrated a high positive GBS rate if dual colonization is tested (vaginal/rectal; 81.1%) and low rate if only vaginal or only rectal samples are tested (13.5, 5.4%, respectively).

It is important to be aware that the included studies were performed in countries with different economic backgrounds, some of them being undeveloped. This might have influenced different laboratory possibilities since some used traditional tests only (microscopy and culture), while in nine studies, PCR tests (NAATs) were used [9,13–15,18,19,22,26,27]. Traditionally, very specific culture tests have been used and recommended for GBS detection, but the application of appropriate high-sensitive molecular tests significantly increases detection results [35]. It is well known that various laboratory tests (e.g., cultivation, Ag tests, NAATs) may yield various results [22], because their sensitivity and specificity may vary. Here, we observed a positive rate from 0.2 to 20.8% for conventional tests and from 37 to 45% for molecular tests. Also, the culture and molecular tests exhibit a great difference in positive rate with values of 18 and 49%, respectively, for the same patients [13]. Some studies revealed that the liquid chromogenic medium has a high specificity (98.1%) and coincidence rate, much higher than chromogenic agar recommended by the CDC (70.6%) [29]. Therefore, to increase the possibility of detecting the causative agent, enriched broth culture is often used in conjunction with traditional agar plate cultures, especially when low levels of GBS are expected in the sample. To overcome this limitation, there is a suggestion to include differential chromogen plates, which are incubated under anaerobic conditions, and data showed that this method increases sensitivity [5]. However, globally, culture-based testing is still predominate due to the cost, laboratory equipment, and test specificity. Thus, this is recommended by the guideline for GBS differentiation as a standard protocol, but in this survey, the suggested method has been rarely used. It is known that culture tests are still the gold standard in microbiological laboratories, especially since they allow accurate microbial identification, susceptibility testing, and serotyping. For the accurate identification a new proteomic method, such as MALDI-TOF MS, is promising [36], but this tool is only available in developed countries. Therefore, only a single study in this survey reported it [17].

Nevertheless, Ag tests or NAATs (PCR) have become more attractive due to short performance time and higher sensitivity. Matter of fact, their laboratory performance and clinical utility are still under investigation, but preliminary data are promising. GBS Ag detection test was found to be more sensitive than the standard tests done by culture. It has a low cost and can be performed in basic diagnostic microbiology services with the potential to replace the standard culture for screening for GBS [28]. The disadvantage of this method is that susceptibility testing is not possible, as well as serotyping or identification of culture. In the field of microbial detection, a significant focus is on NAATs, but when designing the molecular-based detection test, the biological and genetic diversity of GBS, which is relatively large, should be taken into consideration. The literature review showed divergent results regarding GBS screening and test performance. Nevertheless, the evaluation of the analytical performances of NAATs GBS screening is limited and should be highlighted.

Despite screening options and significant progress in early laboratory detection of GBS, EONI including neonatal sepsis is still the third major cause of neonatal deaths resulting in 203,000 deaths per year [37], and the recent data showed that the risk of neonatal sepsis was 5.45 times higher in women who were screened positive when compared to non-GBS carriers [38]. However, it is important to note that other gram-positive bacteria (coagulase-negative *Staphylococcus*, *S. aureus*, *Streptococcus pneumonia*, *Enterococcus*), gram-negative bacteria (*E. coli, Klebsiella pneumoniae*, *Acinetobacter baumannii*), and some yeasts (e.g., *Candida*) are emerging neonatal sepsis pathogens [2,37], so with the present survey it is highlighted that several studies demonstrated vaginal colonization with microbes other than GBS (Table 1). Despite the fact that high adherence to GBS screening recommendations and using an intepartum NAAT gives highly sensitive results, with the ability to significantly reduce the likelihood of neonatal infections, from the future perspective in the prevention of GBS and EONI in general, new microbiological tests, new clinical prediction models and risks estimation, and new monitoring strategies seems crucial [39]. From a laboratory perspective, this primarily means including point-of-care tests, multiplex specific PCR tests, and tests combining differential agar for polymicrobial detection for screening vaginal colonization during pregnancy are able to detect all pathogens which may be potentially involved in EONI [29,30,40]. Following these recommendations and steps, our study group has organized an interactive platform for “prediction, prevention, and personalization in microbiology” in order to reduce the likelihood of neonatal infection or development of EONI, and ongoing studies based on professional and patient education and participation in the diagnosis of selected infections in pregnancy and implementation of novel platform have been designed.

## Supplementary Material

Supplementary Table

## References

[1] Pangerl S, Sundin D, Geraghty S. Group B Streptococcus screening guidelines in pregnancy: a critical review of compliance. Matern Child Health J. 2021 Feb;25(2):257–67. 10.1007/s10995-020-03113-z. Epub 2021 Jan 4. PMID: 33394277. 33394277

[2] Simonsen KA, Anderson-Berry AL, Delair SF, Davies HD. Early onset neonatal sepsis. Clin Microbiol Rev. 2014;27:21–47. 10.1128/CMR.00031-13. PMC3910904 24396135

[3] Verani JR, McGee L, Schrag SJ. Division of Bacterial Diseases, National Center for Immunization and Respiratory Diseases, Centers for Disease Control and Prevention (CDC). Prevention of perinatal group B streptococcal disease–revised guidelines from CDC, 2010. MMWR Recomm Rep. 2010;59(RR-10):1–36. 21088663

[4] Prevention of group B streptococcal early-onset disease in newborns: ACOG committee opinion summary, number 782. Obstet Gynecol. 2019;134(1):1. 10.1097/AOG.0000000000003335. 31241596

[5] Filkins L, Hauser JR, Robinson-Dunn B, Tibbetts R, Boyanton BL, Revell P. American society for microbiology provides 2020 guidelines for detection and identification of group B Streptococcus. J Clin Microbiol. 2020;59(1):e01230–20. 10.1128/JCM.01230-20. PMC7771461 33115849

[6] Page MJ, McKenzie JE, Bossuyt PM, Boutron I, Hoffmann TC, Mulrow CD, et al. The PRISMA 2020 statement: an updated guideline for reporting systematic reviews. BMJ. 2021;372:n71. 10.1136/bmj.n71. PMC8005924 33782057

[7] Moola S, Munn Z, Tufanaru C, Aromataris E, Sears K, Sfetcu R, et al. Chapter 7: Systematic reviews of etiology and risk. In: Aromataris E, Lockwood C, Porritt K, Pilla B, Jordan Z, editors. JBI manual for evidence synthesis. London, UK: JBI; 2020.

[8] Arena B, Daccò MD. Evaluation of vaginal microbiota in women admitted to the hospital for premature labor. Acta Biomed. 2021;92(5):e2021292. 10.23750/abm.v92i5.9925. PMC8689340 34738595

[9] De Francesco MA, Caracciolo S, Gargiulo F, Manca N. Phenotypes, genotypes, serotypes and molecular epidemiology of erythromycin-resistant Streptococcus agalactiae in Italy. Eur J Clin Microbiol Infect Dis. 2012;31(8):1741–7. 10.1007/s10096-011-1495-4. 22120421

[10] Stokholm J, Schjørring S, Eskildsen CE, Pedersen L, Bischoff AL, Følsgaard N, et al. Antibiotic use during pregnancy alters the commensal vaginal microbiota. Clin Microbiol Infect. 2014;20(7):629–35. 10.1111/1469-0691.12411. 24118384

[11] Tazi A, Doloy A, Réglier-Poupet H, Hemet ME, Raymond J, Poyart C. Evaluation du nouveau milieu chromogène StrepB Select pour le dépistage anténatal des streptocoques du groupe B chez la femme enceinte [Evaluation of the new chromogenic medium StrepB Select for screening of group B Streptococcus in pregnant women]. Pathol Biol (Paris). 2009;57(3):225–8. 10.1016/j.patbio.2008.09.002. 19008052

[12] Dechen TC, Sumit K, Ranabir P. Correlates of vaginal colonization with group B Streptococci among pregnant women. J Glob Infect Dis. 2010;2(3):236–41. 10.4103/0974-777X.68536. PMC2946679 20927284

[13] Dilrukshi GN, Kottahachchi J, Dissanayake DMBT, Pathiraja RP, Karunasingha J, Sampath MKA, et al. Group B streptococcus colonization and their antimicrobial susceptibility among pregnant women attending antenatal clinics in tertiary care hospitals in the Western Province of Sri Lanka. J Obstet Gynaecol. 2021;41(1):1–6. 10.1080/01443615.2020.1716313. 32172646

[14] Kan H, He Y, Li Q, Mu Y, Dong Y, Fan W, et al. Differential effect of vaginal microbiota on spontaneous preterm birth among Chinese pregnant women. Biomed Res Int. 2022;2022:3536108. 10.1155/2022/3536108. PMC9731763 36506912

[15] Morozumi M, Chiba N, Igarashi Y, Mitsuhashi N, Wajima T, Iwata S, et al. Direct identification of Streptococcus agalactiae and capsular type by real-time PCR in vaginal swabs from pregnant women. J Infect Chemother. 2015;21(1):34–8. 10.1016/j.jiac.2014.08.024. 25287153

[16] Matsubara K, Katayama K, Baba K, Nigami H, Harigaya H. Prevalence of group B streptococcal type VI capsular IgG antibodies in Japan. Eur J Clin Microbiol Infect Dis. 2003;22(7):453–4. 10.1007/s10096-003-0942-2. 12827529

[17] Zhou J, Zhang L, Zhang Y, Liu H, Xu K, Zhang B, et al. Analysis of molecular characteristics of CAMP-negative Streptococcus agalactiae strains. Front Microbiol. 2023;14:1189093. 10.3389/fmicb.2023.1189093. PMC10244789 37293216

[18] Teatero S, Ferrieri P, Martin I, Demczuk W, McGeer A, Fittipaldi N. Serotype distribution, population structure, and antimicrobial resistance of group B Streptococcus strains recovered from colonized pregnant women. J Clin Microbiol. 2017;55(2):412–22. 10.1128/JCM.01615-16. PMC5277510 27852675

[19] Witkin SS, Nasioudis D, Leizer J, Minis E, Boester A, Forney LJ. Epigenetics and the vaginal microbiome: influence of the microbiota on the histone deacetylase level in vaginal epithelial cells from pregnant women. Minerva Ginecol. 2019;71(2):171–5. 10.23736/S0026-4784.18.04322-8. 30318873

[20] Abdelaziz ZA, Ibrahim ME, Bilal NE, Hamid ME. Vaginal infections among pregnant women at Omdurman Maternity Hospital in Khartoum, Sudan. J Infect Dev Ctries. 2014;8(4):490–7. 10.3855/jidc.3197. 24727516

[21] Tumuhamye J, Steinsland H, Tumwine JK, Namugga O, Mukunya D, Bwanga F, et al. Vaginal colonisation of women in labour with potentially pathogenic bacteria: a cross-sectional study at three primary health care facilities in Central Uganda. BMC Infect Dis. 2020;20(1):98. 10.1186/s12879-020-4821-6. PMC6995194 32005177

[22] Shawaky SM, Al Shammari MMA, Sewelliam MS, Ghazal AAER, Amer AN. A study on vaginitis among pregnant and non-pregnant females in Alexandria, Egypt: an unexpected high rate of mixed vaginal infection. AIMS Microbiol. 2022;8(2):167–77. 10.3934/microbiol.2022014. PMC9329880 35974993

[23] Sebitloane HM, Moodley J, Esterhuizen TM. Pathogenic lower genital tract organisms in HIV-infected and uninfected women, and their association with postpartum infectious morbidity. S Afr Med J. 2011;101(7):466–9. 21920099

[24] Tchelougou D, Karou DS, Kpotsra A, Balaka A, Assih M, Bamoke M, et al. Infections vaginales chez les femmes enceintes au centre hospitalier régional de Sokodé (Togo) entre 2010 et 2011 [Vaginal infections in pregnant women at the Regional Hospital of Sokode (Togo) in 2010 and 2011]. Med Sante Trop. 2013;23(1):49–54. 10.1684/mst.2013.0142. 23692693

[25] Ghaddar N, El Roz A, Ghssein G, Ibrahim JN. Emergence of vulvovaginal candidiasis among lebanese pregnant women: prevalence, risk factors, and species distribution. Infect Dis Obstet Gynecol. 2019;2019:5016810. 10.1155/2019/5016810. PMC6699268 31467477

[26] Juliana NCA, Deb S, Juma MH, Poort L, Budding AE, Mbarouk A, et al. The vaginal microbiota composition and genital infections during and after pregnancy among women in Pemba Island, Tanzania. Microorganisms. 2022;10(3):509. 10.3390/microorganisms10030509. PMC8951098 35336085

[27] Mukesi M, Iweriebor BC, Obi LC, Nwodo UU, Moyo SR, Okoh AI. Prevalence and capsular type distribution of Streptococcus agalactiae isolated from pregnant women in Namibia and South Africa. BMC Infect Dis. 2019;19(1):179. 10.1186/s12879-019-3809-6. PMC6383256 30786878

[28] Rallu F, Barriga P, Scrivo C, Martel-Laferrière V, Laferrière C. Sensitivities of antigen detection and PCR assays greatly increased compared to that of the standard culture method for screening for group B streptococcus carriage in pregnant women. J Clin Microbiol. 2006 Mar;44(3):725–8. 10.1128/JCM.44.3.725-728.2006. PMID: 16517846; PMCID: PMC1393163. PMC1393163 16517846

[29] Gao K, Deng Q, Huang L, Chang CY, Zhong H, Xie Y, et al. Diagnostic performance of various methodologies for group B streptococcus screening in pregnant woman in China. Front Cell Infect Microbiol. 2021 May;11:651968. 10.3389/fcimb.2021.651968. PMI D: 34109134; PMCID: PMC8183470. PMC8183470 34109134

[30] Charfi R, Guyonnet C, Untrau M, Giacometti G, Paper T, Poyart C, et al. Performances of two rapid LAMP-based techniques for the intrapartum detection of Group B Streptococcus vaginal colonization. Ann Clin Microbiol Antimicrob. 2024;23:37. 10.1186/s12941-024-00695-2. PMC11046945 38664821

[31] Vieira LL, Perez AV, Machado MM, Kayser ML, Vettori DV, Alegretti AP, et al. Group B streptococcus detection in pregnant women: comparison of qPCR assay, culture, and the Xpert GBS rapid test. BMC Pregnancy Childbirth. 2019;19(1):532. 10.1186/s12884-019-2681-0 PMC6937909 31888631

[32] Tonen-Wolyec Serge S, Otuli NL, Otsatre-Okuti M, Atenyi-Kasemire R, Dupont R, Bélec L. Analytical performances of a point-of-care loop-mediated isothermal amplification assay to detect Group B streptococcus in intrapartum pregnant women living in the Democratic Republic of the Congo. Int J Infect Dis. 2024 May;142:106972. 10.1016/j.ijid.2024.02.015. Epub 2024 Feb 20. PMID: 38387704. 38387704

[33] Berikopoulou MM, Pana A, Liakopoulou-Tsitsipi T, Vlahos NF, Papaevangelou V, Soldatou A. Poor adherence to the screening-based strategy of group B streptococcus despite colonization of pregnant women in Greece. Pathogens. 2021 Apr;10(4):418. 10.3390/pathogens10040418. PMID: 33915970; PMCID: PMC8067163. PMC8067163 33915970

[34] Nadeau HCG, Bisson C, Chen X, Zhao YD, Williams M, Edwards RK. Vaginal–perianal or vaginal–perineal compared with vaginal–rectal culture-based screening for Group B Streptococci (GBS) colonization during the third trimester of pregnancy: a systematic review and meta-analysis. BMC Pregnancy Childbirth. 2022 Mar;22(1):204. 10.1186/s12884-022-04546-w. PMID: 35287615; PMCID: PMC8919537. PMC8919537 35287615

[35] Bogiel T, Depka D, Zalas-Więcek P, Rzepka M, Kruszyńska E, Gospodarek-Komkowska E. Application of the appropriate molecular biology-based method significantly increases the sensitivity of group B streptococcus detection results. J Hosp Infect. 2021 Jun;112:21–6. 10.1016/j.jhin.2021.03.008. Epub 2021 Mar 16. PMID: 33741491. 33741491

[36] Tanno D, Saito K, Ohashi K, Toyokawa M, Yamadera Y, Shimura H. Matrix-assisted laser desorption ionization-time-of-flight mass spectrometry with time-of-flight peak analysis for rapid and accurate detection of group B streptococcus in pregnant women. Microbiol Spectr. 2022 Jun;10(3):e0173221. 10.1128/spectrum.01732-21. Epub 2022 Apr 18. PMID: 35435738; PMCID: PMC9241660. PMC9241660 35435738

[37] Attia Hussein Mahmoud H, Parekh R, Dhandibhotla S, Sai T, Pradhan A, Alugula S, et al. Insight into neonatal sepsis: an overview. Cureus. 2023 Sep;15(9):e45530. 10.7759/cureus.45530. PMID: 37868444; PMCID: PMC10585949. PMC10585949 37868444

[38] Fung TY, Sahota DS. How can we reduce neonatal sepsis after universal group B streptococcus screening? BMC Pregnancy Childbirth. 2024 Sep;24(1):586. 10.1186/s12884-024-06791-7. PMID: 39244582; PMCID: PMC11380416. PMC11380416 39244582

[39] Kosmeri C, Giapros V, Serbis A, Baltogianni M. Application of advanced molecular methods to study early-onset neonatal sepsis. Int J Mol Sci. 2024 Feb;25(4):2258. 10.3390/ijms25042258. PMID: 38396935; PMCID: PMC10889541. PMC10889541 38396935

[40] Oeser C, Pond M, Butcher P, Bedford Russell A, Henneke P, Laing K, et al. PCR for the detection of pathogens in neonatal early onset sepsis. PLoS One. 2020 Jan;15(1):e0226817. 10.1371/journal.pone.0226817. PMID: 31978082; PMCID: PMC6980546. PMC6980546 31978082

